# Around the world in 1.5 million nights

**DOI:** 10.1093/sleep/zsaf119

**Published:** 2025-04-28

**Authors:** Oliva Walch

**Affiliations:** Department of Neurology, University of Michigan, Ann Arbor, MI, USA; Arcascope Inc, Arlington, VA, USA

Consider, for a moment, the cost of a single in-lab sleep study. Multiply that by thirty to get a month’s worth of monitoring. Add the cost of an international flight. Big number, right? Now multiply by 64,847.

Phones and wearables allow us to collect vast sums of data that simply would not have been possible to capture in another era. In the case of Willoughby et al., such advances have allowed for the collection of 1.5 million nights of data from travelers wearing Oura Rings on 64,847 journeys around the world [1]. Such an experiment would have been prohibitively expensive—not to mention a logistical nightmare—to run in a prescriptive way. But by leveraging the trips participants were already going on and paying for themselves, alongside the devices that they already owned, Willoughby et al. have arrived at a quantification of sleep during time zone-crossing travel at a scale never before seen.

Do people rest up extra before a flight? Very rarely and not for very long. Willoughby et al. report sleep onset approximately ten minutes earlier than baseline for long eastward trips and not much else for all other trips. A shame, since they also report (as you might have guessed) that sleep suffers significantly on planes, especially on long eastward flights which often stretch overnight.

The rule of thumb is that it takes 1–1.5 days of adjustment for every hour of time zones you cross [2]? Well, maybe not in practice: Willoughby et al. find that sleep timing has still not normalized to pre-travel times even after 15 days in the new destination. This, they suggest, maybe not so much a sign that our understanding of entrainment’s timescale is wrong as it is a reflection of the fact that most people who spend 15 days in a new location are vacationers, and vacationers are going to sleep when they want. Said another way, when freed from the demands of work/life constraints at home, it does not appear that people try very hard to entrain to the schedule they were on under those work/life constraints.

Instead, travelers quickly catch up on sleep duration—within about 2 days, their sleep duration has normalized to within 12 minutes of baseline levels—and gradually relax their sleep onset timing towards approximately 20 minutes *earlier* than baseline for long westward trips and approximately 30 minutes *later* than baseline for eastward trips by day 15. Remarkably, for eastward trips, the long timescale behavior does not seem to care if you crossed three time zones or eight: by day 10 in the new time zone, you have locked into a sleep onset consistently later than where you started. To underscore the point: it is not just that people sleep in when given the chance. Long and short westward travelers, at day 15, are still consistently sleeping and rising *earlier* than they were before their trip.

This result, along with others in Willoughby et al., reveals are in many ways in which we are the architects of our sleep diversity. It’s not just the sun interacting with your genes that determines your chronotype. Under work and home life conditions, we self-select into more extreme patterns of sleep–wake behavior. Take those work and home life conditions away, and we see, similar to what is reported in [3] and [4], that the sleep habits in Willoughby et al. homogenize. Long sleepers become shorter and shorter sleepers become longer. Early sleepers become later and later sleepers become earlier. The widespread of sleep phenotypes observed in modern life have been made substantially wider by the countless ways society informs our choice to turn off the light and/or get into bed. The Munich Chronotype Questionnaire characterizes people by their midsleep time on free days [5]. Perhaps a future version, leveraging wearables like the Oura Ring, will characterize people by their sleep on a proper vacation.

Of course, Oura is not polysomnography. (Would you even *want* polysomnography if you are trying to capture naturalistic sleep under travel conditions?) It is also not a classic “research grade” wearable [6]. Yet in addition to validating the device against the gold standard [7], Willoughby et al. go a step further. By getting Oura to agree to freeze their algorithm for the duration of the data collection period, the authors handle the most pressing concern about validated-but-black-box consumer wearable algorithms: the fear that the algorithm could change midway through the study, irrevocably muddling the results. And while an open-source algorithm could provide even greater insights into the nuances of how sleep was captured in this study, let us not let seeking perfection stand in the way of appreciating the data we have here.

What do we get out of this data? Testable hypotheses, for one. Are people slow to return to baseline sleep timing because they are not pushing themselves to stay up/wake up early on vacation, or is it more driven by light exposure during the day, or is it something else? Is the homogenization in sleep habits driven more by freedom from work (and having to get up early), by disconnection from social habits (not staying up late gaming), by changes in sleep environment (black-out curtains in the hotel), or something else? Could people take a bit of that good (or at least, normalized) sleep on vacation home with them?

We can also put some teeth into our recommendations for travelers. They are not sleeping very much before traveling, and this may be because they genuinely are not able to. But for anyone who *is* able to, we can warn them that this is most important for long eastward trips, where sleep duration on the day of travel drops by more than an hour. We can target young people, who are especially affected by lost sleep. We can prepare long travelers for more wakes during the night and give them a timetable by which they should be feeling better. With future analysis of this dataset, we can recommend the best flight times for people based on their wearable data and warn them of trips that may be particularly tough for them.

Data collections of this kind are only going to get more substantive (tracking meals, light, drug intake etc.) and more illuminating. They are also going to get more massive. As you read this, thousands of people are streaming out of their homes and into airports for honeymoons, getaways, and family vacations. The world is generating an enormous corpus of self-funded free-living phase-shifting protocols that we can now tap into. Let us make sure we do.
